# Detection and analysis of wheat spikes using Convolutional Neural Networks

**DOI:** 10.1186/s13007-018-0366-8

**Published:** 2018-11-15

**Authors:** Md Mehedi Hasan, Joshua P. Chopin, Hamid Laga, Stanley J. Miklavcic

**Affiliations:** 1 Phenomics and Bioinformatics Research Centre, University of South Australia, Mawson Lakes, Adelaide, 5095 Australia; 2 School of Engineering and Information Technology, Murdoch University, Perth, Western Australia 6150 Australia

**Keywords:** Plant phenotyping, Spike detection, Deep learning, Field imaging, Statistical analysis

## Abstract

**Background:**

Field phenotyping by remote sensing has received increased interest in recent years with the possibility of achieving high-throughput analysis of crop fields. Along with the various technological developments, the application of machine learning methods for image analysis has enhanced the potential for quantitative assessment of a multitude of crop traits. For wheat breeding purposes, assessing the production of wheat spikes, as the grain-bearing organ, is a useful proxy measure of grain production. Thus, being able to detect and characterize spikes from images of wheat fields is an essential component in a wheat breeding pipeline for the selection of high yielding varieties.

**Results:**

We have applied a deep learning approach to accurately detect, count and analyze wheat spikes for yield estimation. We have tested the approach on a set of images of wheat field trial comprising 10 varieties subjected to three fertilizer treatments. The images have been captured over one season, using high definition RGB cameras mounted on a land-based imaging platform, and viewing the wheat plots from an oblique angle. A subset of in-field images has been accurately labeled by manually annotating all the spike regions. This annotated dataset, called SPIKE, is then used to train four region-based Convolutional Neural Networks (R-CNN) which take, as input, images of wheat plots, and accurately detect and count spike regions in each plot. The CNNs also output the spike density and a classification probability for each plot. Using the same R-CNN architecture, four different models were generated based on four different datasets of training and testing images captured at various growth stages. Despite the challenging field imaging conditions, e.g., variable illumination conditions, high spike occlusion, and complex background, the four R-CNN models achieve an average detection accuracy ranging from 88 to $94\%$ across different sets of test images. The most robust R-CNN model, which achieved the highest accuracy, is then selected to study the variation in spike production over 10 wheat varieties and three treatments. The SPIKE dataset and the trained CNN are the main contributions of this paper.

**Conclusion:**

With the availability of good training datasets such us the SPIKE dataset proposed in this article, deep learning techniques can achieve high accuracy in detecting and counting spikes from complex wheat field images. The proposed robust R-CNN model, which has been trained on spike images captured during different growth stages, is optimized for application to a wider variety of field scenarios. It accurately quantifies the differences in yield produced by the 10 varieties we have studied, and their respective responses to fertilizer treatment. We have also observed that the other R-CNN models exhibit more specialized performances. The data set and the R-CNN model, which we make publicly available, have the potential to greatly benefit plant breeders by facilitating the high throughput selection of high yielding varieties.

**Electronic supplementary material:**

The online version of this article (10.1186/s13007-018-0366-8) contains supplementary material, which is available to authorized users.

## Background

Wheat is one of the most globally significant crop species with an annual worldwide grain production of 700 million tonnes [1]. In recent years, however, there is an increasing demand for grain. At the same time, the seasonal fluctuations, the extreme weather events and the altering climate in various regions of the world, increase the risk of inconsistent supply. This points to the need to identify hardier and higher yielding plant varieties to both increase crop production and improve plant tolerance to biotic and abiotic stresses.

To discover higher-yielding and more stress-tolerant varieties, biologists and breeders rely more and more on high-throughput phenotyping techniques to measure various plant traits, which in turn are used to understand plant’s response to various environmental conditions and treatments, with the hope to improve grain yield.

Early works on high-throughput image-based phenotyping focused on controlled environments such as purpose-built chambers and automated glasshouses. Li et al. [2], for example, proposed an approach that detects, counts and measures the geometric properties of spikes of a single plant grown in a controlled environment. Bi et al. [3, 4] and Pound et al. [5], on the other hand, measured more detailed morphological properties, such as the numbers of awns and spikelets, of plants imaged in small purpose-built chambers with uniform backgrounds. Unfortunately, in such experiments plants are confined to small pots, which no doubt affect root development, nutrient uptake and, ultimately, yield. Some experiments have been carried out using plants grown in large (120 cm $\times$ 80 cm) indoor bins, which are capable of housing almost 100 plants in competition [6–8]. Spike detection was not attempted in these latter studies, but their more critical limitation was that the plants, although grown closer to field-like conditions and not individually in pots, were not subject to realistic environmental conditions. The challenge to providing quantitative plant breeder support is yield estimation under true field conditions, relying on the ability to accurately and automatically detect and count the ears of wheat in the field.

A range of different phenotyping platforms exist for capturing images in the field [9–11]. However, due to the large scale nature of such studies, many researchers have turned to aerial imaging systems such as unmanned aerial vehicles [12–15] and satellite imagery [16, 17]. While these approaches are capable of capturing information about a large number of plants across a large area of land within a short period of time, only coarse level information, such as mean canopy coverage and mean canopy color, has thus far been reported. It should also be kept in mind that the nature of the uncontrolled field environment poses significant challenges for both image acquisition and image analysis algorithms, which should ideally be robust to changing conditions and applied autonomously. The challenges indeed often result in images being analyzed manually or semi-automatically, and often qualitatively.

In this study we utilize a land-based vehicle and a single RGB camera to acquire images of a field. The proximity of the camera to the plants allows for high-resolution data capture. The simplicity of the imaging set-up makes it affordable and easy to implement, thus accessible to any potential user. The remaining challenge, on which we focus attention here, is of analyzing these high resolution images to extract quantitative information such as the number and density of wheat spikes. To go some way to meeting this challenge we have chosen to image plots from an oblique perspective as opposed to the more common nadir perspective. In an oblique view a significant number of spike features such as texture, color, shape etc. can be discerned easily. These features can be more readily extracted for the purposes of various plant phenotyping applications such as spike counting (which is the focus of this paper), spike shape measurement, spike texture, disease detection, grain yield estimation etc. We note that we are not unique in taking this more advantageous perspective [7, 18, 19].

There are some computer vision approaches for detecting spikes in field images obtained using land-based imaging techniques, which have been reported in the literature. Fernandez-Gallego et al. [20] used RGB cameras manually held at approximately one meter above the center of the plant canopy to gain images from a nadir perspective. The authors then apply the Laplacian and the median filter to produce a transformed image where the local maxima can be detected and classified as wheat spikes. This approach achieved a recognition rates of up to 92%, but failed when observing plants in different developmental stages (32%). Alharbi et al. [18], which used Gabor filters, principal component analysis and k-means clustering, were able to achieve an average accuracy of 90.7%. The approach, however, places constraints on image content such as the density of spikes, color and texture differences between spikes and shoots and the angle of spikes in the image. Zhou et al. [21] proposed an image fusion method by using multi-sensor data and an improved maximum entropy segmentation algorithm to detect wheat spikes in the field. However, the method required the use of a multi-spectral camera and was validated on images where canopy and spikes rarely overlap or occlude one another.

Machine learning has been adopted as the method of choice in many recent image analysis applications to address a number of plant phenotyping problems. These include the study of wheat spikes in controlled environments [2], the classification of leaf species and leaf venation [22], the analysis of the architecture of root systems [23, 24], the measurement of plant stress levels [25] and the determination of wheat growth stages [26]. More recently, deep learning has begun to outperform previous image analysis and machine learning approaches and promises a step-change in the performance of image-based phenotyping. In particular, the use of Convolutional Neural Networks (CNNs) for image analysis tasks has seen a rapid increase in popularity. For instance, CNNs have been used to improve the performance of the approach of Wilf et al. [22] for identifying and counting leaf species [27], to quantitatively phenotype *Arabidopsis thaliana* plants grown in controlled environments [28, 29], and to provide detailed quantitative characterization of wheat spikes on plants grown in controlled environments [3–5].

In this study we present the first deep learning model designed specifically to detect and characterize wheat spikes present in wheat field images. We adapt, train and apply a variant of CNN, hereinafter referred to as *Region-based Convolutional Neural Networks (R-CNN)*, to accurately count wheat spikes in images acquired with our land-based RGB imaging platform. The approach relies on a training data set of images containing spikes that have been labeled manually with rectangular boxes; The procedure produces a complete list of locations and dimensions of bounding boxes identifying plant spikes detected in images unseen during the training stage. A successful deep learning analysis requires thorough training using large data sets of high quality [5, 30]. As such, a second major contribution of this work is the release of the SPIKE data set, made up of hundreds of high quality images containing over 20,000 labeled wheat spikes.

The outline of this article is as follows. In the "Methods" section we describe the field trial we have studied and the image acquisition system. The images from this field trial form the SPIKE data set which is then described in detail and used for training and testing of our R-CNN model. Finally, we also present the metrics used for the validation of the proposed CNN model. In the "Results and discussion" section we analyze the performance of the model both on the main data set and on subsets containing images of field plots at different growth stages. We also provide an analysis of the density of spikes detected in images of plots of different wheat varieties treated with fertilizer at different times. In brief, we found that early treatment resulted in significantly higher yields (spike densities) for nearly all the varieties tested, than what were produced by the same varieties either untreated or treated later in the season.

## Methods

**Fig. 1:**
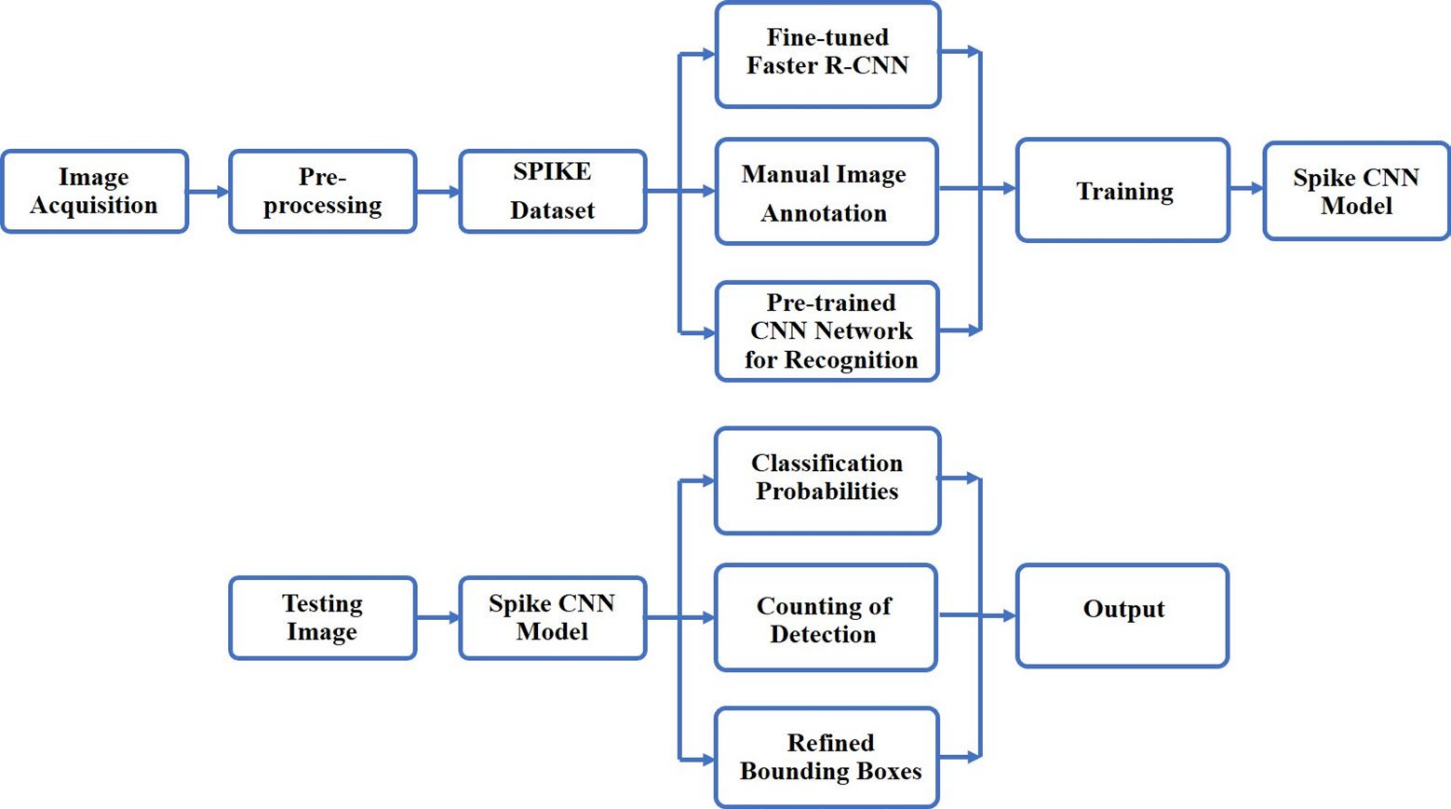
The general work-flow diagram of the proposed system. The top diagram shows the training procedure of the Convoluted Neural Network (CNN) model implemented in this article. The bottom diagram depicts the testing procedure that is followed to obtain our results

Figure 1 shows the overall work-flow of the in-field wheat spike detection system. The goal is to develop a fast and accurate system which can detect spikes from field images. The output is a list of bounding boxes enclosing wheat spikes, as well as the confidence level for each box, along with a count of the total number of spikes. The model has been developed in two main stages: the training stage, used to train the R-CNN for spike detection, and the testing stage, in which the trained CNN model is applied to test images.

### Experimental setup

The field trial was conducted at Mallala (− 34.457062, 138.481487), South Australia, in a randomized complete block design with a total of 90 plots, 18 rows and 5 columns, consisting of ten spring wheat (*Triticum aestivum* L.) varieties (Drysdale, Excalibur, Gladius, Gregory, Kukri, Mace, Magenta, RAC875, Scout, Yitpi) and nine replicates of each, all of which were sown on July 3, 2017. To mitigate the boundary effects, an additional plot (not included in the analysis) was planted at the beginning and at the end of each row of plots. The plots were 1.2 m wide, with an inter-row spacing of approximately 0.2 m, and 4 m long with a gap of approximately 2 m between plot rows and 0.3 m between columns. To explore the impact of fertilizer on wheat spike production, each variety was subject to three fertilizer treatments: no treatment, early treatment, and late treatment. Each combination of variety $\times$ treatment is replicated three times. Two thirds of the replicates were treated at a standard rate of 80 kg nitrogen, 40 kg phosphorus and 40 kg potassium per hectare (referred to as $16-8-16 \; N-P-K$), while the other 30 plots received no treatment at all. For the early treatment, the macronutrients nitrogen, phosphorus and potassium were applied on July 14. Urea was then applied on July 18 to the same 30 plots. For the late stage treatment, both fertilizers were applied together on September 26. The imaging of the plots took place approximately twice a week during the period of July 21–November 22, 2017.

**Fig. 2:**
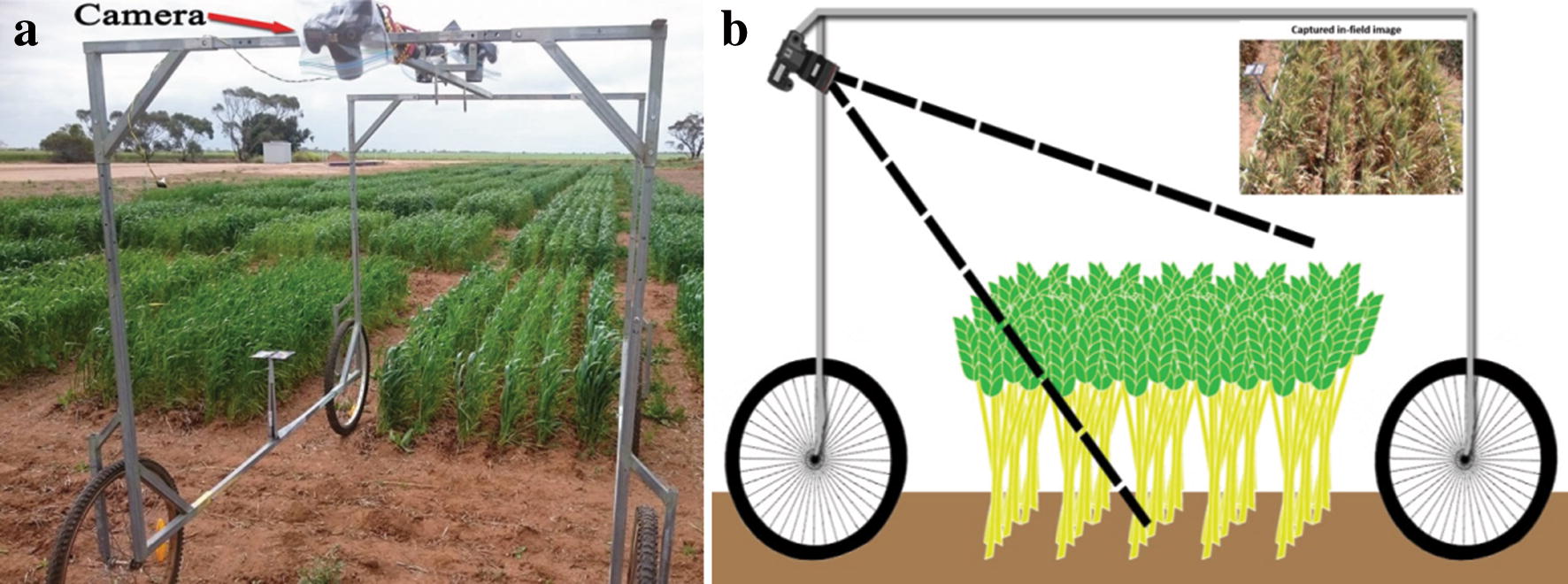
The ground-based vehicle for imaging in the field. **a** A camera, angled for oblique viewing, is placed at the top of an imaging frame mounted on a four-wheel base (the wagon). The frame also supports two stereo cameras, angled vertically, placed in the center of the top section. These have not been used in this article. **b** A schematic of the wagon from a side-view. A sample image taken with the oblique-view camera is shown in the inset, top right

The land-based vehicle used for image capture is shown in Fig. 2. This wagon is comprised of a steel frame and four wheels with a central overhead rail for mounting imaging sensors. While capable of housing a stereo pair of cameras for orthogonal viewing, only the camera mounted at one end at an angle oblique to the plots was used for this study. Viewed from directly above, many spikes, primarily those near the viewing axis, appear in images small and circular, making them difficult to detect (see the comparison of images of the same plot taken from the two perspectives in Additional file 1). Although not pursued in this paper, a perspective view also admits the possibility of a more detailed analysis of spikes (for, say, grain number estimation) with a greater fraction of their length visible, although the problem of partial occlusion of some spikes may complicate the estimation process. Figure 2b (inset, top right) shows an image captured with this imaging platform. The images were acquired using an 18.1 megapixel Canon EOS 60D digital camera, shown in Fig. 2a, surrounded with a waterproof casing. Manual focus was used during all the imaging sessions with the camera focused at 2.2 m and 1.8 m during early and late plant growth stages, respectively. Following some experimentation, a viewing angle of $55^{\circ }$ from the horizontal overhead rail was chosen to capture a maximum plot area with minimal the area from overlapping regions. The camera sensor is located 190 cm above the ground level. The camera settings were as follows;

- Focal length—18 mm,
- Aperture—f/9.0,
- ISO—automatic and
- Exposure time—1/500 s.

Finally, the resolution of images was 5184 $\times$ 3456 pixels, resulting in an image resolution of approximately 0.04 cm per pixel.

### The SPIKE dataset

The high quality in-field images from this field trial are used to construct the SPIKE data set, a key contribution of this study. The SPIKE data set has three main components:

- Over 300 images of ten wheat varieties at three different growth stages.
- Annotations for each image denoting the bounding boxes of spikes.
- Deep learning models trained on these images and labels.

**Fig. 3:**
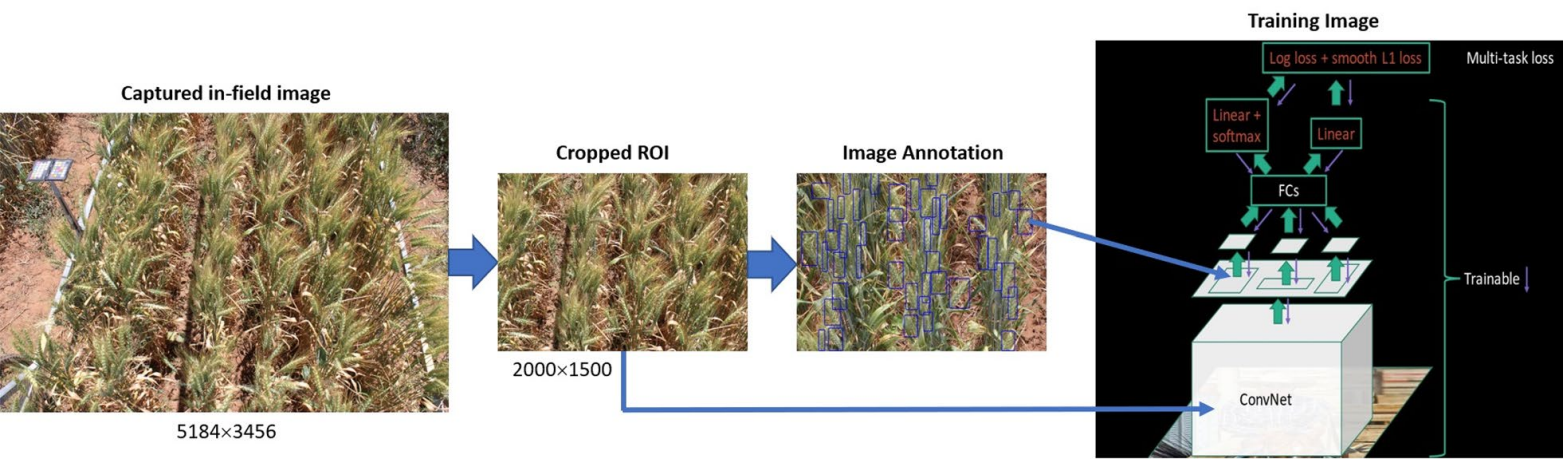
Steps for training the model. Images are acquired in the field before being automatically cropped to a region of interest. Training images were then manually annotated with bounding boxes. Finally, both the cropped and annotated images were passed to the R-CNN model for training

A diagram illustrating each of these components is shown in Fig. 3. First, images are acquired in the field. These are then automatically cropped so that only the region of interest (ROI) is kept. The captured in-field images contain other objects including neighbor plots, plot gaps, vehicle and color-chart which are not required in our approach. So, a significant SPIKE region from the plot is selected as ROI and cropped automatically for all images in the experiment. Next, the images are manually annotated with bounding boxes highlighting all the spikes present in the images. The images and annotations are then fed to the Convolutional Neural Network (CNN) for training.

*Images* While the original images capture the majority of the 4 m $\times$ 1.2 m plot area, they also contain parts of the neighboring plots, inter-plot weeds and parts of the wagon. These background objects can confound the testing phase; a particular issue is spikes of neighboring plots appearing in an image and thus included in the density estimation. To overcome this issue, images were automatically cropped to a 0.8 m $\times$ 0.8 m region of interest as shown in Fig. 3. In total, 335 images containing a total of approximately 25,000 wheat spikes have been captured. With our camera image resolution, the spike size [width, height] ranged from [10 px, 80 px] to [50 px, 300 px].

**Fig. 4:**
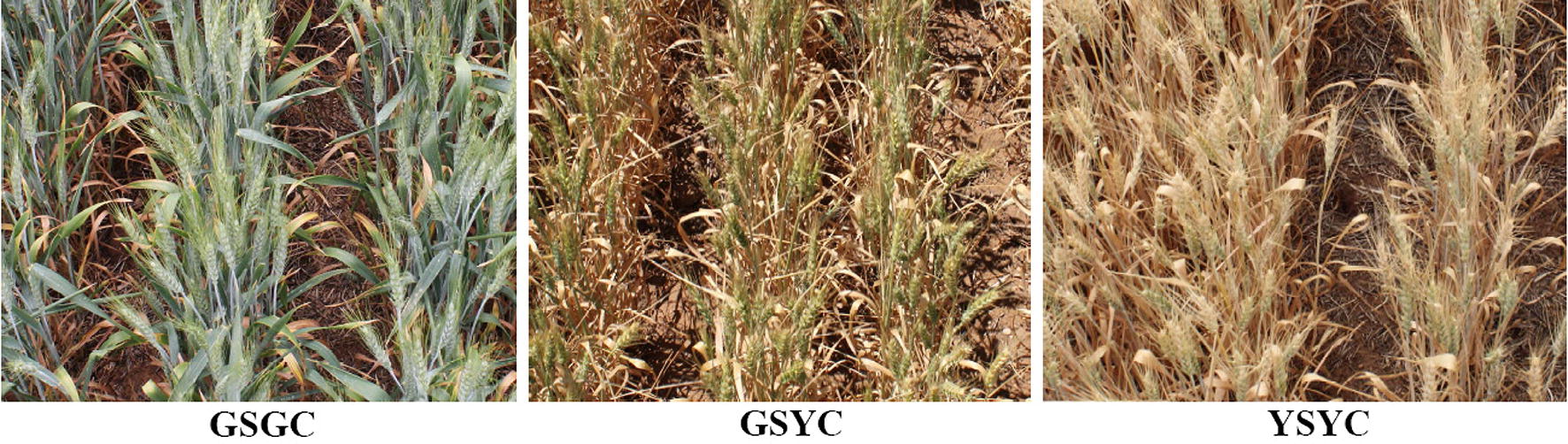
Examples of training images captured at three different growth stages. From left to right: the GSGC images contain green spikes and a green canopy, the GSYC images contain green spikes and a yellow canopy, and the YSYC images contain yellow spikes and a yellow canopy.

We found that the most convenient situation for detecting wheat spikes in images is when there is considerable color contrast between the spikes and other parts of the canopy. As such, the majority of the images in the SPIKE data set contains images where the spikes are approximately green in color while the canopy has already senesced to a more yellow color. However, in order to fully test the capabilities of deep learning techniques for spike detection in the field, the SPIKE data set also includes a number of images taken at two other growth stages, where spike detection spikes is more difficult. Hereafter we denote the three different situations, shown in Fig. 4, as:

- Green Spike and Green Canopy (GSGC)
- Green Spike and Yellow Canopy (GSYC)
- Yellow Spike and Yellow Canopy (YSYC).

The GSGC, GSYC, and YSYC images were acquired on the 26/10/2017, 9/11/2017 and 16/11/2017, respectively. Table 1 shows the number of images acquired for each of the three classes. Although the data set contains 255 GSYC images, only 235 were used for training while the remaining 20 were reserved for testing. Each of the GSGC and YSYC data sets comprise 40 images, of which 35 have been used for training and 5 for testing. The second half of the table, which indicates how many images were used in the different models, will be explained in more detail at the end of this section.

**Table 1:** Number of images from each growth stage used for training and testing

Images	GSYC	GSGC	YSYC	
Data				
Training	235	35	35	
Test	20	5	5	
Total	255	40	40	

Images	GSYC	+ GSGC	+ YSYC	GSYC++
Models				
Training	235	270	270	305
Test	20	25	25	30

**Fig. 5:**
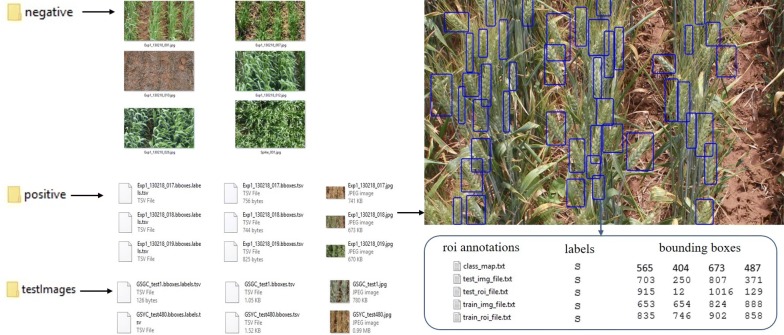
Representation and annotation of the SPIKE data set. The data set is split into three subsets: the positive, negative and test images. Image labeling classes, locations and their annotations are saved in a separate text file

*Annotations* The images have been labeled by multiple experts at the resolution of $2000\times 1500$ pixels. For the annotation of images, we used the publicly available Video Object Tagging Tool provided by Microsoft. Each labelled image has an additional text file containing the coordinates of the annotated bounding boxes, see Fig. 5. In this file the boxes are saved as a 4-tuple $(x_b,y_b,w_b,h_b)$ where $(x_b,y_b)$ denotes the top-left corner of the box while the pair $(w_b,h_b)$ denotes the width and height of the bounding box. Each image contains approximately 70–80 spikes. Therefore, in total, the 335 images contain approximately 25,000 annotated spikes.

*Model development* The SPIKE data set of 335 images in total was split into 305 training images and 30 testing images. This split was performed at the image level, not at the spike level, to ensure that no spikes from the same image could be seen in both training and testing sets. We found that the GSYC class of images, which exhibit a high color contrast, were the most suitable for spike detection in the field. For this reason the main model used in this study was trained and tested only on the set of GSYC images. However, in order to better understand the effect of spike and canopy color on deep learning models we trained three additional models using the two other classes. The reader is referred to the bottom half of Table 1 for a summary of the number of training and testing images used in each of the four models. The + GSGC and + YSYC models were trained using the original 235 images as well as the 35 GSGC images and 35 YSYC images, respectively. They also have a set of test images made up of combinations of the test images from their corresponding classes. Finally, a fourth model, ‘GSYC++’, was based on the 305 training images from all three classes and had a test set comprised of all the 30 designated test images.

### R-CNN model

Region-based Convolutional Neural Network (R-CNN) was introduced by Girshick et al. [31] for object detection using a selective search to detect regions of interest and a CNN to classify them. Later, Fast R-CNN by ROI pooling [32] was used after final convolution to extract a fixed length feature vector from the feature map along with the training of all network weights with back-propagation. Later, Faster R-CNN was developed by Ren et al.  [33]. This model consists of two networks: a region proposal network (RPN) for generating region proposals, and a convolutional network which takes the proposed regions to detect objects almost in real-time. The main difference between the two region-based methods is that, to generate region proposals, Fast R-CNN uses selective search whereas Faster R-CNN uses high-speed RPN and shares the bulk of the computation time with object detection. Briefly, RPN ranks the region boxes (called anchors) and proposes the ones that are most likely to contain the desired objects. Due to its fast processing capability and high recognition rate, Faster R-CNN is used in this article for wheat spike detection. Python implementation of the Faster-RCNN is publicly available and can be accessed online [34]. The implementation is modified somewhat and hyper-parameters have been optimized for better classification of the spike regions and overall detection performance. A detailed description of R-CNN, the specific architecture of the model, and the image processing techniques used in this article can be found in Additional file 2.

For each box detected, the R-CNN provides as output a corresponding confidence level, $C\in [0,1]$, where 0 represents the lowest level of confidence that a detected object is a spike and 1 represents the highest level of confidence. When a detected box proposed by the CNN has a confidence value *C* that is larger than a predefined threshold, then the proposal is classified as a spike. Otherwise, it is classified as a background. Higher values of *C* will result in fewer boxes being incorrectly labeled as spikes, but will also result in more spikes being incorrectly labeled as background. Conversely, low values of *C* will correspond to incorrectly captured (background) regions but will rarely miss plant spikes. In this study we have chosen to use a confidence value of $C=0.5$ as it provided a desirable trade-off between the two scenarios.

### Validation

The output of the R-CNN used in this study is a list of bounding boxes which will ideally contain all of the wheat spikes in an image. The goal of this study is for the number of boxes to accurately match the number of spikes in an image. Denoting boxes as spike or non-spike can yield three potential results, with the latter two being sources of error: *true positive (TP)*—correctly classifying a region as a spike; *false positive (FP)*—incorrectly classifying a background region as a spike as well as multiple detection of the same spike; and *false negative (FN)*—incorrectly classifying a spike as a background region. In contrast, *true negative (TN)*—correct classification of background is always ’zero’ and is not required in this binary classification problem where foreground is always determined for object detection. In order to quantify our errors, the validation metrics are based on the concepts of precision, recall, accuracy and the F1 score, which are defined as follows:

- $\text {Precision} = \displaystyle \frac{TP}{TP+FP}$ measures how many of the detected regions are actually spikes.
- $\text {Recall} = \displaystyle \frac{TP}{TP+FN}$ measures how many of the spikes in the image have been captured.
- $\text {Accuracy} = \displaystyle \frac{TP+TN}{TP+TN+FP+FN}$ implies the models performance
- $\text {F1 Score} = \displaystyle 2 \frac{Precision\cdot Recall}{Precision + Recall}$ is the harmonic mean of Precision and Recall. It is a useful measure to observe a model’s robustness.
- The mean Average Precision (mAP) [35], which quantifies how precise the method is at varying levels of Recall. It can be expressed as follows:
  1 $$\begin{aligned} \begin{aligned} mAP = \frac{1}{11}\sum _{r_{i}\in \left\{ 0,0.1,...,1\right\} }^{}\max _{r_{i}:r_{i}\ge r}p(r_{i}). \end{aligned} \end{aligned}$$
  In other words, it is defined as the mean precision of a set of eleven equally spaced Recall levels $[0,0.1,\ldots ,1]$. Here, $p(r_i)$ is the measured Precision at Recall $r_i$. The Precision at each Recall level $r_i$ is interpolated by taking the maximum Precision measured for which the corresponding Recall exceeds *r*.

All the experiments in this article were conducted using a high-performance computer with Intel Xeon 3.50 GHz processor and 128 GB of memory. Also, a NVIDIA GeForce graphics processing unit(GPU) has 12 GB memory which is used along with the CPU to accelerate the training of the CNN.

## Results and discussion

The performance of the proposed model was measured in terms of detection accuracy and mean precision defined in the Validation Section. To demonstrate the robustness of deep learning for spike detection, we analyzed the degrees to which the different training and testing data sets, captured at different growth stages, affect the model performance. Finally, we analyze the differences in spike density across the different varieties grown under the three different treatments in the field trial.

### Performance

**Fig. 6:**
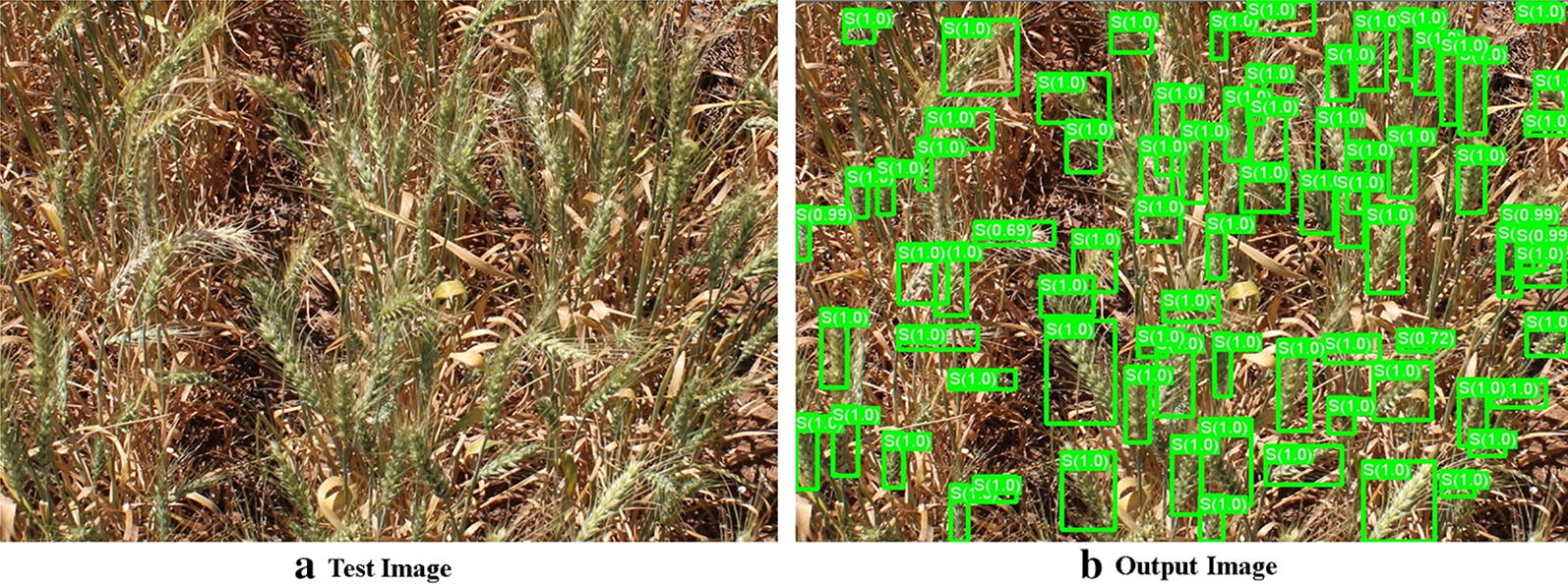
An example of a generated output image (**b**) from a test image (**a**). Detected spikes are indicated using bounding boxes along with their respective classification confidences. Among 82, 78 spikes were detected with a mAP of 0.7211 and an accuracy of 95.18%

For each test image the R-CNN program returns the locations of the detected spikes, the total number of spikes, and a classification probability (confidence) for each detected spike, see Fig. 6. The GSYC class of images was chosen to train the main model proposed in this study. For 20 test images, the model achieved a mAP of 0.6653 and an average accuracy of $93.3\%$ based on the 1463 spikes detected among the 1570 manually counted spikes. For each test image, the following statistics are provided in Table 2; the number of spikes in the ground truth image, the number of spikes detected by the proposed approach, the number of true positives, the number of false positives, the number of false negatives, the precision, the mAP, the accuracy, and the F1 score. The output images of this table are included in the supplementary material (Additional file 2).

**Table 2:** Evaluation and validation of spike detection using the GSYC image model applied to the GSYC image data set

Image no	GT-count	Detected	TP	FP	FN	Precision	mAP	Accuracy	F1-score
Test_001.jpg	73	71	70	1	3	0.98	*0.7289*	*96*%	0.97
Test_012.jpg	75	68	68	0	7	1.00	*0.6002*	*91*%	0.95
Test_025.jpg	87	85	84	1	3	0.98	*0.7324*	*96*%	0.97
Test_032.jpg	80	76	76	0	4	1.00	*0.7286*	*95*%	0.97
Test_118.jpg	76	73	70	3	6	0.95	*0.6126*	*92*%	0.93
Test_141.jpg	66	61	58	3	8	0.95	*0.5835*	*88*%	0.91
Test_185.jpg	69	68	65	3	4	0.95	*0.7105*	*94*%	0.94
Test_199.jpg	72	69	68	1	4	0.98	*0.7184*	*94*%	0.96
Test_220.jpg	80	79	76	3	4	0.96	*0.7229*	*95*%	0.95
Test_242.jpg	70	64	63	1	7	0.90	*0.5926*	*90*%	0.94
Test_254.jpg	83	77	76	1	7	0.98	*0.6085*	*91*%	0.95
Test_320.jpg	80	77	74	3	6	0.96	*0.6213*	*92*%	0.94
Test_383.jpg	87	84	78	6	9	0.92	*0.5947*	*90*%	0.91
Test_399.jpg	80	78	77	1	3	0.98	*0.7301*	*96*%	0.97
Test_417.jpg	96	93	89	4	7	0.95	*0.6573*	*93*%	0.94
Test_421.jpg	71	73	70	3	1	0.95	*0.7552*	*98*%	0.97
Test_422.jpg	82	79	78	1	4	0.98	*0.7211*	*95*%	0.96
Test_432.jpg	85	81	79	2	6	0.97	*0.6502*	*93*%	0.95
Test_437.jpg	70	64	62	2	8	0.96	*0.5924*	*88*%	0.92
Test_480.jpg	88	84	82	2	6	0.97	*0.6441*	*93*%	0.95
Total	1570	1504	1463	41	107	−	−	−	−
Average	−	−	−	−	−	*0.97*	*0.6653*	*93.4*%	*0.95*
Standard dev.	7.82	8.17	7.86	1.46	1.11	0.02	0.06	0.03	0.02

### Testing the supplementary models

In this section, the results of the base GSYC model are compared with those of the other three models. The comparative analysis for different testing sample combinations is presented in Table 3 in terms of the average detection accuracy (ADA) and in Table 4 in terms of mean Average Precision.

**Table 3:** average detection accuracy (ADA) ($\%$) of the Faster R-CNN on different SPIKE dataset models

Test images	Base	Extended model		
	GSYC (%)	+ GSGC (%)	+ YSYC (%)	GSYC++ (%)
GSYC (20)	93.4	92.2	91.7	92.9
GSGC (5)	89.6	94.5	87.4	93.7
YSYC (5)	84.8	86.5	93.1	92.3
GSYC + GSGC + YSYC (30)	89.8	90.7	91.9	93.2

**Fig. 7:**
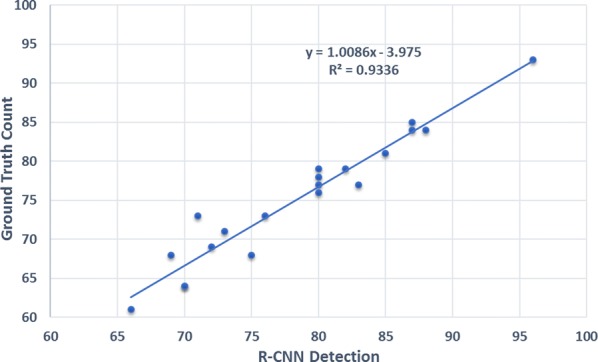
Ground truth versus estimated number of spikes per plot. The horizontal axis refers to the number of spikes estimated by the proposed approach and the vertical axis refers to the number of spikes that have been manually counted

From Table 2, one can see that the spike detection accuracy is always within the range of 88–98 for the 20 images tested. This is quite satisfactory considering the challenges associated with in-field imaging, e.g., complex backgrounds, various illumination conditions, shadow effects and self occlusion. Also, the high mAP of 0.6653 shows the proficiency of our R-CNN, trained on the SPIKE data set. This is to be compared with the mAP performance of other CNN’s applied to prominent data sets such as PASCAL VOC  [35] and COCO [33], for the detection of 21 and 80, respectively, regular object classes such as, men, car, horse, dog, cat, bicycle, etc. Figure 7 shows the relationship between ground truth number of spikes and the estimated number of spikes, for each of the 20 images. The R-CNN approach provides a near one-to-one estimate of the number of spikes per image (the line slope is 1.0086), with an intercept value of $-\,3.95$ indicating an intrinsic error of just four spikes. The model produces a high $R^2$ value of 0.93, proving a strong linear relationship between the ground truth and the results of our approach.

**Fig. 8:**
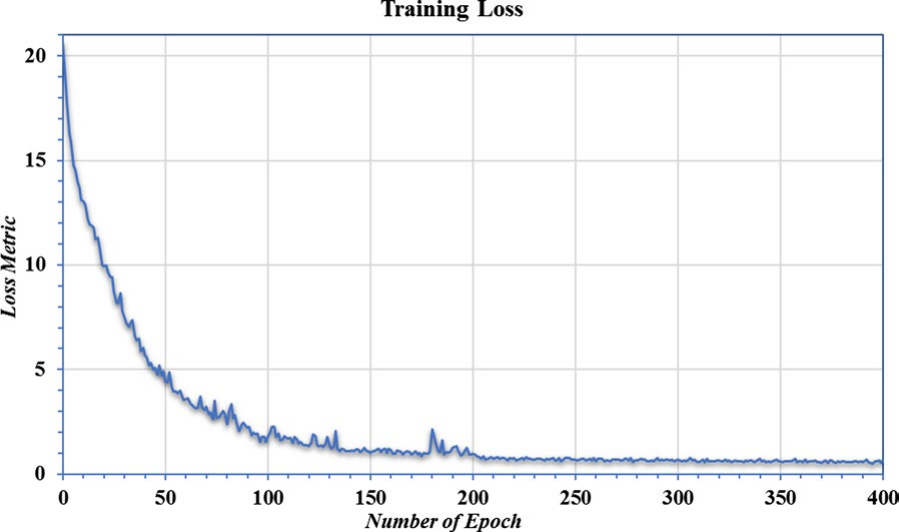
Number of epochs versus training loss. While training loss begins large it steadily decreases over the training epochs until eventually, at around 200 epochs, the benefit of further training appears to be negligible

The efficiency of a training model can also be analyzed by observing the training loss and error rates while the model is learning. An epoch is defined as one full pass forwards and backwards through the network during the learning stage. While the model weights are initialized randomly, after a number of epochs they become closer to their final values, progressively reducing rates of error and training loss. Figure 8 shows that the loss metric (described in full detail in Additional file 2) is decreasing over subsequent epochs of training. Although the loss and error rate is initially high, after each training epoch the reduced rate of error is accompanied by a higher detection accuracy; the loss and error rates become almost constant after 200 epochs indicating that no further improvement is possible. Based on several trials the number of epochs was fixed at 400 to avoid overfitting. This choice produced the high accuracy results presented in this article.

When limited to GSYC images, the GSYC model proved to return the highest accuracy in terms of ADA, valued as a percentage of spikes detected, as testing and training images covered plants at the same growth. When applied to GSGC or YSYC testing images, however, while still achieving a high accuracy, the performance had declined. Including GSGC and YSYC images in the test image set reduced the accuracy from 93.4 to 91.8% and 88.7%, respectively. Clearly, detection accuracy deteriorates when testing with images that are unknown to the trained model. Note also that the lower detection accuracy following inclusion of YSYC images in the GSYC data set points to the increased difficulty of differentiating yellow spikes from yellow canopy. The ADA comparison reflects the anticipated and indeed intuitive fact that a model can perform best when applied to similar types of images as those used for training. The consistent mAP results confirm the ADA finding.

The same situation is reflected by the + GSGC and + YSYC models. These models work well when applied to image types that are included in the respective training sets. Not surprisingly, the GSYC++ model performs consistently better, in terms of both ADA and mAP, for all types of testing samples. It is not clear what factors are responsible for the highest degree of accuracy found for the GSYC + YSYC + GSGC image set. In light of the superior accuracy of the GSYC++ model it can be concluded that a model is particularly robust if trained with all types of spike-versus-canopy scenarios. With no a priori knowledge of samples, this model will perform better than the other training models. In fact, in the other models, the mAP for spike detection is reduced wherein GSYC++ model it is higher while maintaining the higher accuracy of $93.2\%$. Considering that we are dealing with in-field imaging complexities and we are seeking to detect hundreds of spikes in an image, the mAP value of 0.6763 leading to a $93.2\%$ detection accuracy with the extended GSYC++ model is significantly better than the performance exhibited with the conventional VOC07 or COCO data sets  [33], with values ranging from 64 to $78\%$.

From Tables 3 and 4, it can be concluded that if a model is trained properly, Faster R-CNN can detect with high accuracy spikes in images that were acquired at the same growth stage and in an equivalent category. The precision of a model may drop but its scalability and robustness will depend on how well it is trained, particularly by including all different types of complex scenarios. Based on the performances of the different CNN models and considering the ADA and mAP metrics for bounding box regression described in Additional file 2, the $GSYC++$ model was chosen to analyze the spike density variation across the different treatments applied to the different wheat varieties. For this latter investigation we selected an imaging date that is different from the dates used for data acquisition and training of the CNN models.

**Table 4:** Detection mAP of Faster R-CNN on different SPIKE dataset models

Test images	Base	Extended model		
	GSYC	+ GSGC	+ YSYC	GSYC++
GSYC (20)	0.6653	0.6462	0.6435	0.6575
GSGC (5)	0.6570	0.7077	0.6405	0.6857
YSYC (5)	0.6546	0.6590	0.7163	0.7085
GSYC + GSGC + YSYC (30)	0.6050	0.6413	0.6520	0.6763

### Spike density analysis

A third contribution of this paper is a comparative analysis of spike density for the different wheat varieties under the different treatments. The 10 varieties underwent three different fertilizer treatments: no treatment, early treatment, and late treatment. Determining spike density as a function of genotype and treatment should provide some insight into their relative contribution to yield. The latter is based on the total number of detected spikes within the ROI within each plot, resulting in an estimate of spike density (number per square meter). Since the ROI is uniformly cropped and consistently defined, edge effects are minimized. To quantify spike density, we have constructed another test set different from the set of images used in training and from the previous testing analysis. The image set is derived from the imaging session conducted on 7/11/2017. This test set contains 90 images of the 10 different varieties subject to the three treatments, with three replicates for each case. We remark in passing that the spike densities found in this study were consistent with the conditions for the region and standard sowing rate (45 g of seed per plot). The densities thus are not as high as found in other parts of Australia or elsewhere in the world.

**Fig. 9:**
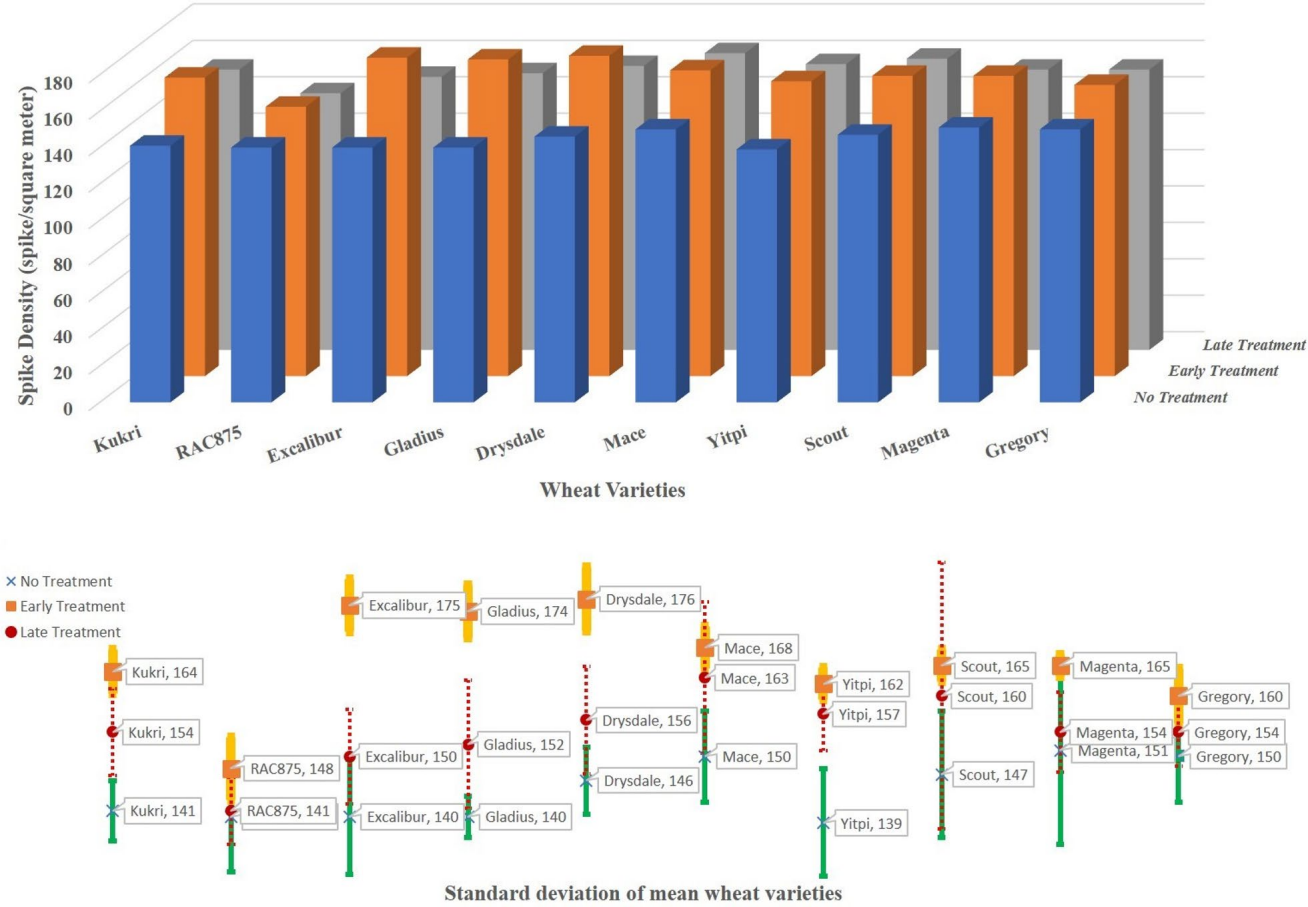
Spike density for different varieties and treatments. Varieties are sorted by highest spike density under early treatment conditions. Blue bars represent spike density averaged over plots with no treatment, orange bars are averaged over plots with early treatment and grey bars are averaged over plots with late treatment. The bottom graph shows the standard deviation of mean values of different wheat varieties for various types of treatments. Red dotted, orange solid and green solid bars show, respectively, the standard deviation for late, early and no treatment of varieties

Table 5 shows the number of spikes detected using the GSYC++ model. For the different categories of variety $\times$ treatment, the average values show the mean number of spikes detected in the three replicated plots. It is clear that the untreated wheat plants generally produced fewer spikes per square meter compared with either of the other two treatments. In the case of early fertilization, the varieties Excalibur, Drysdale and Gladius produced significantly more spikes (and hence greater spike densities) than the other varieties (see Fig. 9). The effect of an early treatment was more moderate for Kukri, Mace and Scout, whose densities increased by just over 15 spikes per square meter. In complete contrast, the effect of fertilizer application on RAC875, at either time point, was negligible.

**Table 5:** Spike density (per square meter) of wheat varieties for different types of treatments. The detection is performed using the GSYC++ model

Wheat	No treatment				Early treatment				Late treatment			
Varieties	Rep1	Rep2	Rep3	Avg	Rep1	Rep1	Rep3	Avg	Rep1	Rep2	Rep3	Avg
Kukri	136	146	141	141	165	160	167	164	148	162	152	154
RAC875	130	148	142	140	154	145	145	148	135	146	142	141
Excalibur	145	129	146	140	173	180	172	175	145	159	146	150
Gladius	142	142	136	140	169	177	176	174	140	156	160	152
Drysdale	152	141	145	146	170	178	180	176	159	146	163	156
Mace	158	143	149	150	170	170	164	168	173	167	149	163
Yitpi	146	129	142	139	160	161	165	162	150	160	161	157
Scout	135	152	154	147	163	164	168	165	139	158	183	160
Magenta	133	160	160	151	166	163	166	165	160	147	155	154
Gregory	149	158	143	150	161	164	155	160	149	160	153	154

Regarding the timing of treatment, the early stage treatment resulted in significantly higher yields for nearly all varieties than what was produced by the same variety treated later in the season. We speculate that this was due at least in part to the longer exposure time of the fertilized soil to rainfall, which facilitated greater uptake of nutrients than possibly occurred with the plants treated later in the season. On the other hand, it is also possible that the comparison is simply consistent with established findings [36] that an early treatment results in greater biomass, while a later treatment can instead result in increased grain nitrogen content. Unfortunately, no analysis of the grain was conducted in this field trial to confirm such an outcome. Further studies are underway to assess the importance of timing on the question of grain filling versus biomass production.

Shown also in Fig. 9 is the degree of variation between replicates of the 10 cultivars under different treatments. In the majority of cases, adding fertilizer early in the season reduced the degree of variation across replicates: no treatment resulted in a deviation of between 3 and 15 $\hbox {spikes/m}^2$ over the 10 varieties, while for the plots treated early, the spread reduced to between 1 and 5 $\hbox {spikes/m}^2$. The greater consistency possibly highlights another aspect of fertilizer treatment. Applying fertilizer later in the season did little to improve consistency, with only 2 out of 3 replicates showing similar results, the third differing significantly, as found in the case of no treatment. Indeed, if one removes the outliers then one could conclude that, as in the case of RAC875, there is little difference between the untreated plots and the late treated plots of Gregory, Excalibur and Magenta.

## Conclusion

Estimating the yield of cereal crops grown in the field is a challenging task, yet it is an essential focus of plant breeders for wheat variety selection and improved crop productivity. Most of the previous works involving image analysis of wheat spikes have been conducted in laboratory conditions and controlled environments. Here, we have presented the first deep learning models for spike detection, trained on wheat images taken in the field. The models are capable of accurately detecting wheat spikes within a complex and changing imaging environment. The best performing model produced an average accuracy and F1 score of $93.4\%$ and 0.95, respectively, when tested on 20 images containing 1570 spikes in total. Although we have not applied the model to oblique-view images of higher spike density field plots, due to the lack of access to such images, we expect the model to perform well at higher densities notwithstanding partial occlusion. Improvement is nevertheless possible by complementing the SPIKE data set with further training images of partial spike objects. The ability to count spikes in the field, a trait closely related to crop yield, to such a degree of accuracy, without destructive sampling or time consuming manual effort, is a significant step forward in field-based plant phenotyping.

## Additional file

**Additional file 1.** View Comparison and Spike Detection Results Comparison between images captured from the top and oblique view angle. Additional spike detection results which contain the original image and corresponding spike detected output image for GSGC, GSYC and YSYC test images.

**Additional file 2.** CNN for Spike Detection. Technical details of the overall Faster R-CNN architecture and step-wise description to train the model for spike detection.
