# Correction to: Detection and analysis of wheat spikes using Convolutional Neural Networks

**DOI:** 10.1186/s13007-019-0405-0

**Published:** 2019-03-20

**Authors:** Md Mehedi Hasan, Joshua P. Chopin, Hamid Laga, Stanley J. Miklavcic

**Affiliations:** 1 Phenomics and Bioinformatics Research Centre, University of South Australia, Mawson Lakes, Adelaide, 5095 Australia; 2 School of Engineering and Information Technology, Murdoch University, Perth, Western Australia 6150 Australia

## Correction to: Plant Methods (2018) 14:100 10.1186/s13007-018-0366-8

In the original publication of this article [1] the authors stated that important resources would be made available online to readers. Unfortunately, due to an error on the authors' behalf, a link to those resources was not included in the final version of the manuscript.

The SPIKE dataset, including images of wheat spikes and labelled bounding boxes for individual spikes, as well as the four trained CNN models described in the article, can now be found at the following sourceforge page:

https://sourceforge.net/projects/spike-dataset/

The link provides access to the free download of all images used in the article as well as their ground truth labelling. The four CNN models available for download at the sourceforge link are to be used in conjunction with the microsoft cognitive toolkit, available at:

https://docs.microsoft.com/en-gb/cognitive-toolkit/

Finally, an additional more user-friendly program has also been uploaded to the sourceforge page. This program contains the same functionality as is currently available through the cognitive toolkit. However, the user will be able to quickly and easily select a dataset of their own images and run the models for spike detection, without downloading and installing the cognitive toolkit.
