# Supplementary material for: Detection and analysis of wheat spikes using Convolutional Neural Networks
Source: Plant Methods. 2018 Nov 15;14:100. doi: 10.1186/s13007-018-0366-8 (PMC6236889; doi:10.1186/s13007-018-0366-8)
Supplement: Supplementary file 1 — Additional file 1. View Comparison and Spike Detection Results Comparison between images captured from the top and oblique view angle. Additional spike detection results which contain the original image and corresponding spike detected output image for GSGC, GSYC and YSYC test images. [file 13007_2018_366_MOESM1_ESM.pdf]

Detection and Analysis of Wheat Spikes using Convolutional Neural Networks

Additional File 1: View Comparison and Spike Detection Results

Md Mehedi Hasan, Joshua P. Chopin, Hamid Laga, Stanley J. Miklavcic

Comparison between Top and Oblique View Image

In the comparison we consider images of the same plot area taken at the same time (from the GSYC image dataset) but taken two different angles, from directly above and from our oblique perspective as shown in Figure S1.1:

Top View: In the image taken from directly above the plot, we counted a total of 183 spikes. Among these, only 120 spikes are sufficiently visible in terms of their shape and texture, while the remaining 63 spikes appear as small regions or as circular tips (exemplified by the red circles in the top image). As these 63 spikes are hard to detect, training a network to detect these regions in addition to spikes visible longitudinally with their distinctly different features, is challenging; the resulting disjoint cluster of classes will likely result in higher error rates in the overall classification process compared with a single cluster of information. The situation with GSGC or YSYC images, in which spikes have the same color as the overall canopy, is even more challenging.

Oblique View: In contrast to the above, a total of 180 spikes were detected in this image of the same plot captured from an oblique perspective. Although some are only partially visible due to overlap with other spikes and leaves, the visible parts have features that are consistent with the training scheme and are therefore still possible to detect. Of the observed spikes, 90% are visible in full length which greatly facilitates detection as well as the physical characterization of the spike regions.

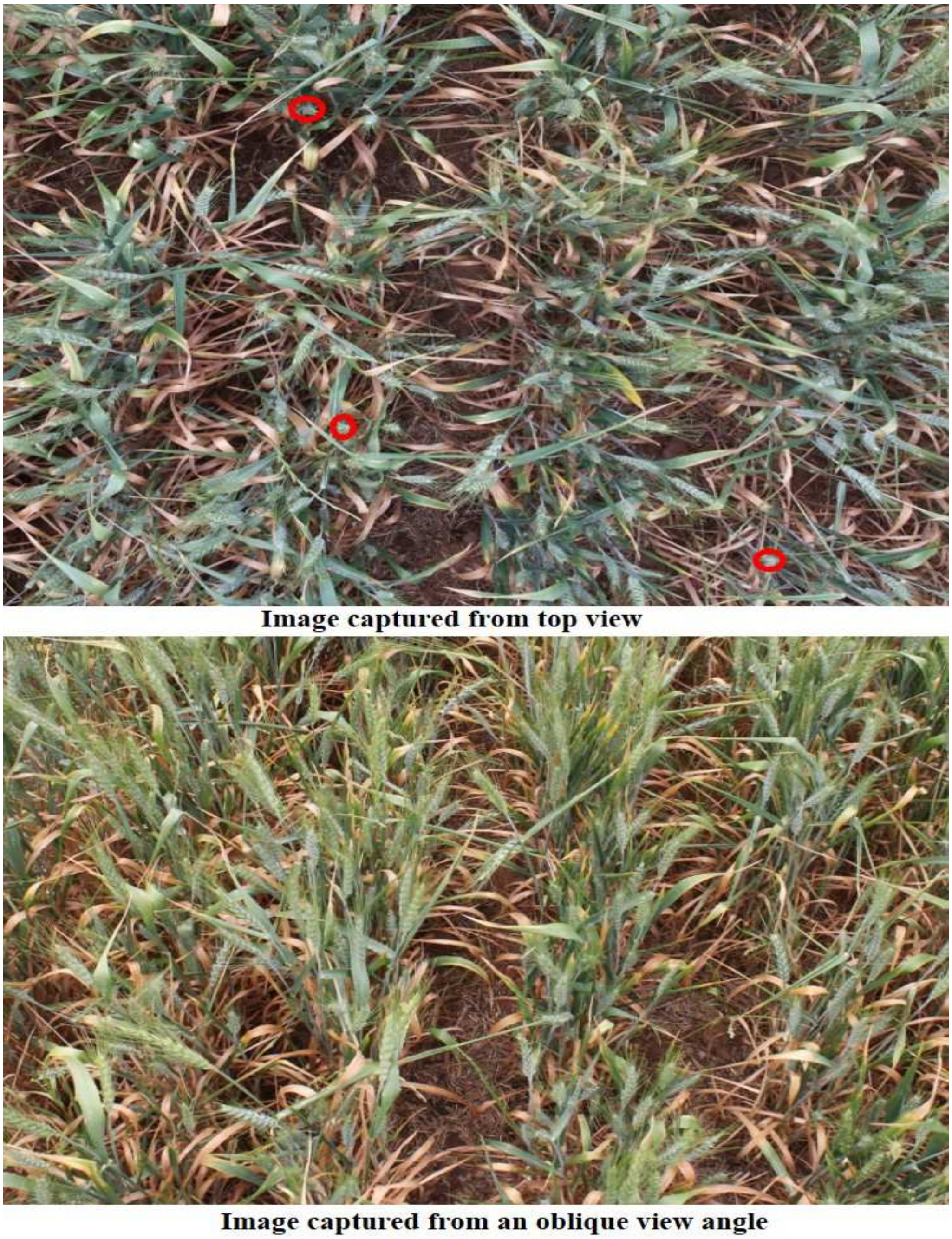

Figure S1.1 GSYC image captured in top view and oblique view angle. Same plot area is investigated for comparative analysis.

Spike Detection Results

The original oblique view input images and the corresponding spike detection results obtained using the GSGC, GSYC and YSYC image datasets are shown in Figure S1.2, Figure S1.3 and Figure S1.4, respectively.

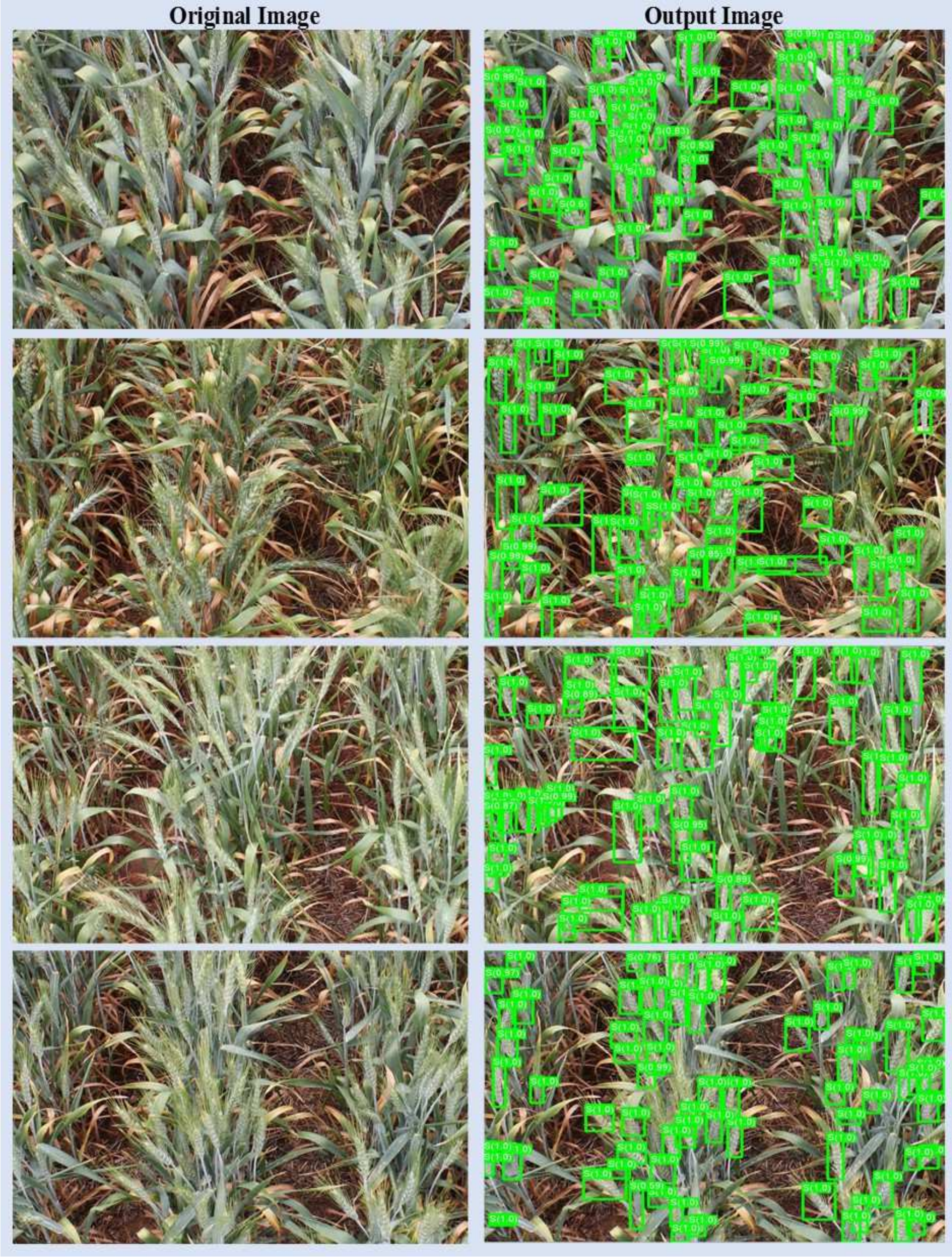

Figure S1.2 The original input images and the corresponding spike detect results obtained using the GSGC image dataset.

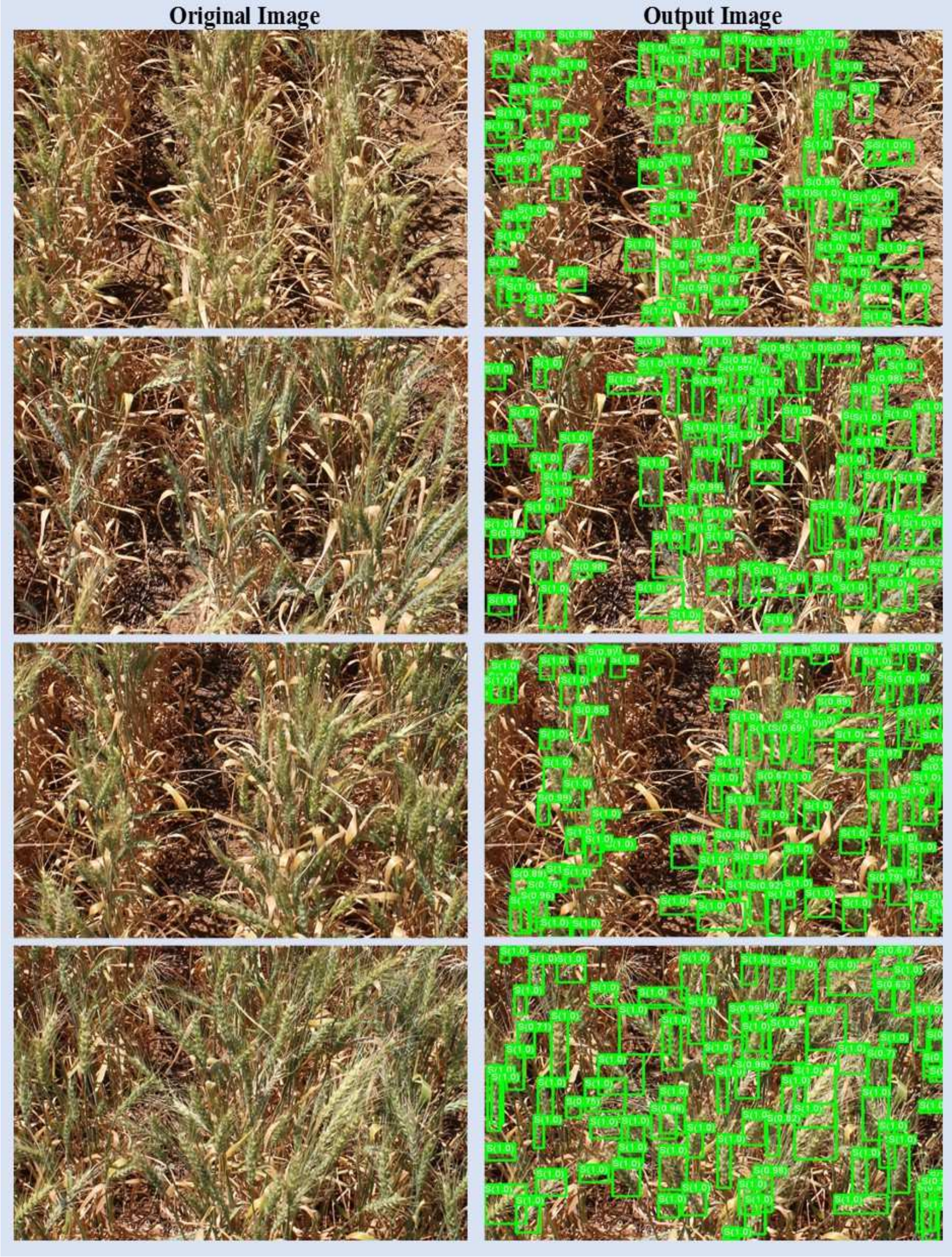

Figure S1.3 The original input images and the corresponding spike detect results obtained using the GSYC image dataset.

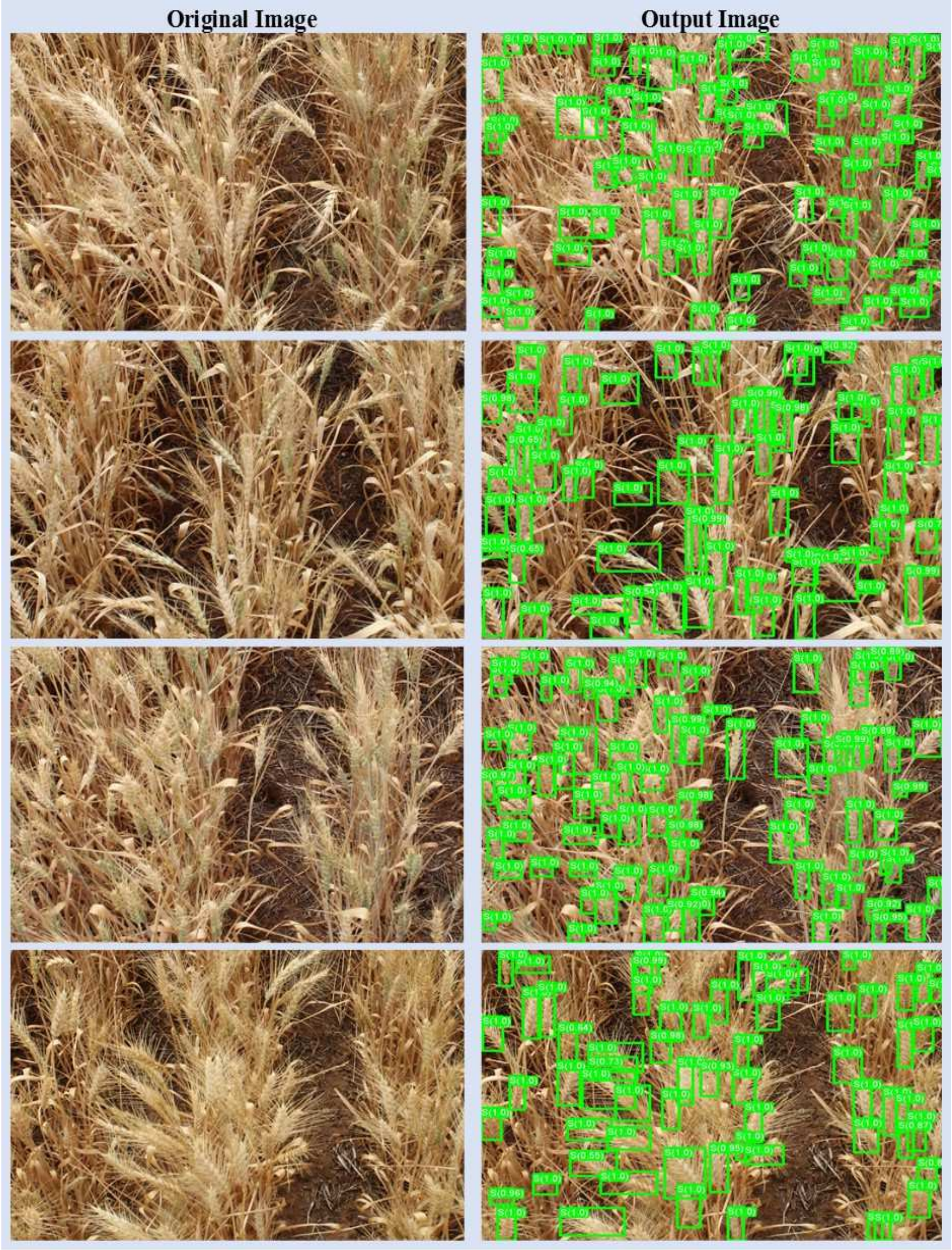

Figure S1.4 The original input images and the corresponding spike detect results obtained using the YSYC image dataset.
