# Supplementary material for: Detection and analysis of wheat spikes using Convolutional Neural Networks
Source: Plant Methods. 2018 Nov 15;14:100. doi: 10.1186/s13007-018-0366-8 (PMC6236889; doi:10.1186/s13007-018-0366-8)
Supplement: Supplementary file 2 — Additional file 2. CNN for Spike Detection. Technical details of the overall Faster R-CNN architecture and step-wise description to train the model for spike detection. [file 13007_2018_366_MOESM2_ESM.pdf]

Detection and Analysis of Wheat Spikes using Convolutional Neural Networks

Additional File 2: CNN for Spike Detection

Md Mehedi Hasan, Joshua P. Chopin, Hamid Laga, Stanley J. Miklavcic

In our approach, a Faster R-CNN is applied which uses neural networks to solve the spike detection problem by considering three key factors:

1 Identify promising regions from an input image that are likely to contain the spike regions as foreground objects.
2 Compute the object class probability of each spike region. The spike regions with the highest probability is selected as the classification result.
3 Determine the total number of spikes detected to measure density.

Faster R-CNN Architechture

Faster R-CNN consists of three main networks as follows:

1 Convolution Neural Network (CNN)
2 Region Proposal Network (RPN)
3 Classification Network

Figure S2.1 shows the schematic diagram of the Faster R-CNN architecture composed of overall three networks used for spike detection.

The first few layers are initialized from a pre-trained network constitute the CNN as ”head” network. The convolutional feature maps produced by this network are then passed through the RPN which uses a series of convolutional and fully connected layers to produce promising regions that are likely to contain spike regions showed as bounding boxes (key factor-1 mentioned above). These identified regions are then used to cropped out (ROI Pooling) corresponding regions from the feature maps produced by the CNN which are then forwarded to a classification network to classify the spike regions contained in each bounding boxes. The spikes with higher classification probability is considered as detected and then total amount is counted for further processing. Below, we describe in details its main components.

Pre-trained Network

The spike detected problem is experimented with several pre-trained networks as below to determine the best possible CNN.

- AlexNet [1], which has 5 shareable convolution layers,

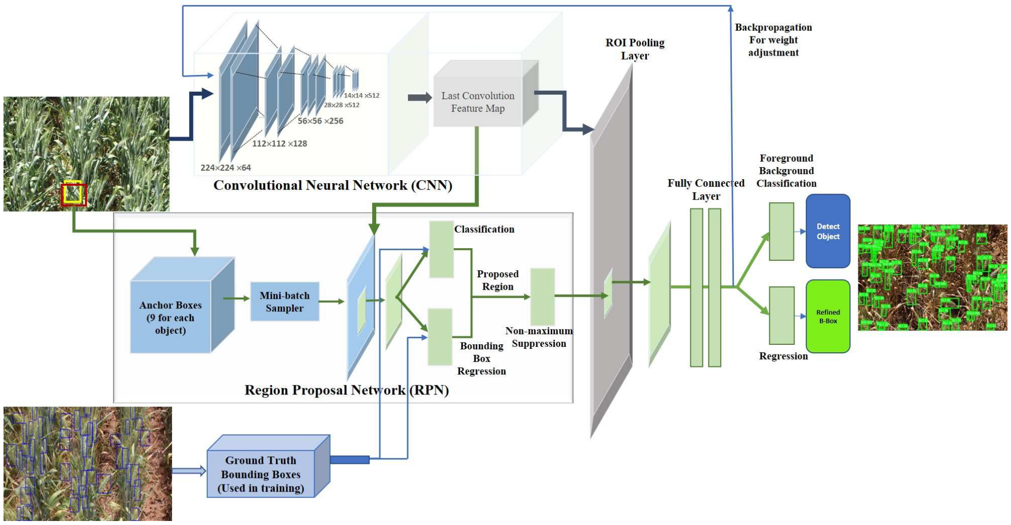

Figure S2.1 The schematic architecture of the Faster R-CNN used for spike detection. Region Proposal Network is generating the region proposals from the anchor boxes and feature map to detect objects.

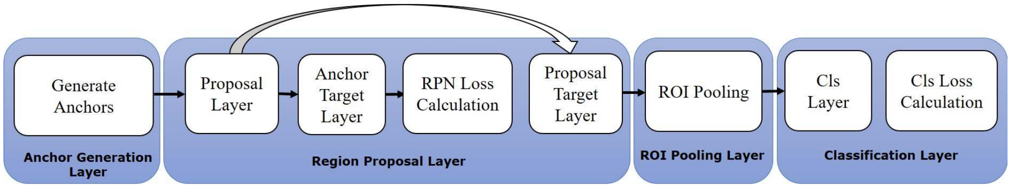

Figure S2.2 R-CNN execution consists of several intermediate layers.

- VGG-16 [2], which has 13 shareable convolutional layers, and
- VGG-19 [2], which has 16 shareable convolutional layers.

Among them, VGG-16 is selected for our spike detection task since it is known to achieve a high object detection and recognition performance on most commonly used open-access datasets [3, 4] as well as for our spike detection task. VGG-16 is known to perform well on tasks such as feature detection in a complex scenarios even when using small training sets [5]. The network takes pre-computed proposals from images and classifies them into object categories and regresses a box around them.

R-CNN execution can be broken down to several intermediate layers as shown in Figure S2.2.

Anchor Generation Layer

Anchors are basically bounding boxes which play an important role for RPN. An anchor has a center, which is also the center of the sliding window, a scale, and an aspect ratio. An

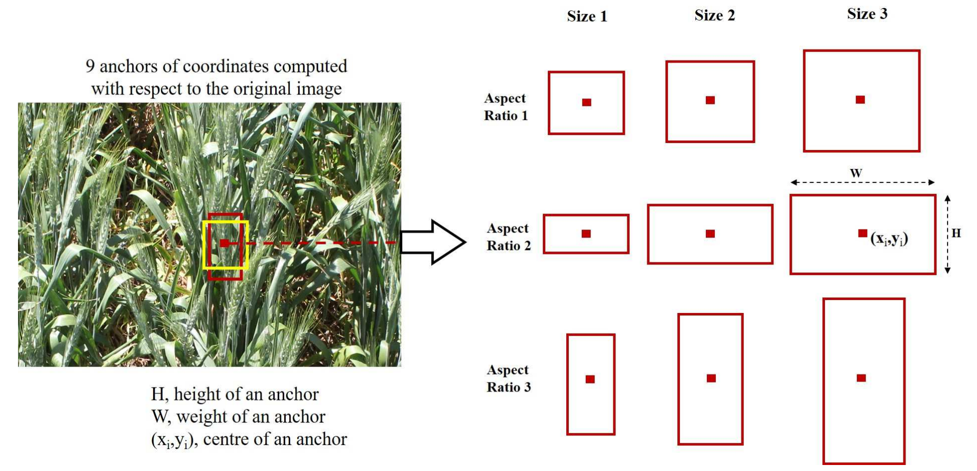

Figure S2.3 Nine anchor boxes proposed to compare with ground-truth bounding boxes. Note that all these coordinates of anchor boxes are computed with respect to the original image.

anchor generation layer produces a set of bounding boxes of varying size and aspect ratios spread all over the input image. In our experiments, we define a set of 9 anchors at each position (x_i, y_i) of an image i by setting three scales and three aspect ratios as shown in Figure S2.3.

Region Proposal Layer

A Region Proposal Network takes as input image, which can be of various sizes, and returns for various regions of the image a score which corresponds to the probability that the region being a spike (foreground) or not a spike, e.g., canopy or soil (background). Region proposal layer transforms the anchors according to the bounding box regression coefficients to generate transformed anchors which are then re-adjusted by applying non-maximum suppression threshold to the probability of an anchor being a foreground region. The anchors generated earlier are forwarded to the region proposal layer as shown in Figure S2.2. In this experiment, considering the small spike regions in an image, anchor proposal scales are set to 2, 4 and 8 and aspect ratios are adjusted as 0.5, 1 and 2.

RPN is constructed with an $n \times n$ convolutional layer followed by two 1×1 convolutional layers (for reg and cls). This is explained in detail in the training section of Faster-RCNN. The goal of the anchor target layer is to produce a set of promising anchors and the corresponding foreground/background labels and apply regression coefficients to train the RPN. Promising foreground anchors are those whose overlap with any ground-truth bounding box is higher than a threshold. In our experiment for each of the anchors, probability p_i^* is computed to show its overlap with the ground-truth bounding boxes as follows:

$$p_i^* = \begin{cases} 1 & \text{if } IoU \geq 0.7, \\ 0 & \text{if } IoU < 0.7. \end{cases} \tag{1}$$

Here, IoU is Intersection over Union, a commonly used overlap measure [6] based on pixel regions and is defined as follows;

$$IoU = \frac{Area\ of\ Overlap}{Area\ of\ Union} = \frac{Anchor \bigcap GTBox}{Anchor \bigcup GTBox}. \tag{2}$$

In RPN the goal of the region proposal layer is to generate good bounding boxes. To do so from a set of anchor boxes, the RPN layer must learn to classify an anchor box as background or foreground and calculate the regression coefficients to modify the position, width and height of a foreground anchor box to make it a "better" foreground box (fit a foreground object more closely). The anchor target layer also generates a set of bounding box regressors as output. Regressor is actually a measure of how far each anchor target is from the closest foreground bounding box. The process of calculating bounding box regression loss and classification loss to generate RPN loss is described later in loss function subsection.

To generate region proposals of spikes, we slide a window over the convolutional feature map generated by the last shared (fc6) layer of CNN followed by RELU. This layer takes as input, an $n \times n$ window of the convolutional feature map which is run through (1,1) kernel convolutional layer to mapped to a lower-dimensional feature . This feature is fed into two fully connected layers- B-box regression and Classification layer. To estimate class probabilities of being spike or not, two kernels and to generate output bounding box regressor four kernel convolution layers have been applied.

For each sliding-window location, we predict multiple spike region proposals for proposal target layer with the maximum number of proposal for each location is denoted as k. Now, the regression layer has $4k$ outputs encoding the coordinates of k boxes and classification which is actually a two-class softmax layer outputs $2k$ scores to estimate the probability of spike or non-spike for each proposal. The scores are parameterized relative to k reference boxes, known as *anchors*. We fine-tuned a pre-trained Faster-RCNN for training with RPN foreground fraction of 0.5 to facilitate maximum number of foreground examples along with the batch size of 256. Batch size defines the number of samples that going to be propagated through the network. To reduce the number of overlapping bounding boxes we applied a non-maximum threshold of 0.7 to the RPN proposal to keep the top 2000 boxes as RPN proposals and the values are determined after several trials to get better accuracy.

ROI Pooling Layer

The Fast R-CNN detector places an ROI pooling layer after the final convolution. It takes mini-batch of region proposals as input. After RPN, we get proposed regions as feature maps of different sizes. The region of interest pooling simplifies these sizes by making the feature maps having the same size. The pooling layer takes the ROI boxes output by the proposal target layer and the convolutional feature maps output by the CNN and outputs square feature maps. The feature maps are then fed the fully connected layer (7×7 in fc6 for VGG-16). The result is a one-dimensional feature vector for each ROI.

Classification Layer

The classification layer takes the output feature maps produced by the ROI Pooling Layer and passes them through a series of convolutional layers. The output is fed through two fully connected layers. The first layer produces the class probability distribution for each region proposal and the second layer produces a set of class specific bounding box regressors. For example, an input image of size 2000×1500 shrinks 16 times to a 125×95 feature map after applying CNNs. Every map contains 9 anchors classified either spike or non-spike. All these feature map and classification label is contained in a feature vector map called *logit*, which is passed to a softmax and a regression layer to predict and classify the regions and the corresponding bounding boxes respectively. Similar to RPN loss, classification loss is the metric that is minimized during optimization to train the classification network. During back propagation, the error gradients flow to the RPN network as well, so training the classification layer modifies the weights of the RPN network as well. The process of calculating bounding box regression loss and classification loss in this layer is described later in loss function subsection.

Training Faster R-CNN

The RPN training minibatch consists of 256 proposals from one image with a $1:1$ ratio of positives (IoU with a ground-truth box larger than 0.7) to negatives (IoU smaller than 0.3). The Fast R-CNN training minibatch has 128 proposals with a $5:1$ ratio of foreground (IoU with a ground-truth box of the same class larger than 0.2) to background ($IoU > 0.7$, with any foreground ground-truth box < 0.2 based on several trials to achieve better accuracy in detection and mAP. During the implementation, we considered three anchor ratios, namely $1:1$, $1:2$, $2:1$. Also, around 12000 anchors are considered during the training process, but by fixing NMS to 0.7 we decrease these proposals to approximately 2000 by taking the top $2k$ proposals used to train R-CNN network. For testing, 600 top scoring boxes are kept after applying NMS 0.8 based on different trials to fulfil the requirement of our approach, which is then used for applying bounding boxes to the detected objects.

Training the overall network requires estimating the weights and minimizing the loss function [6]. Faster R-CNN minimizes a weighted sum of four loss functions:
(1) softmax loss for presence/absence of a foreground object in the RPN,
(2) smooth L1 loss for anchor co-ordinate regression in RPN,
(3) softmax loss for the classification of region proposals for detection require object, and
(4) bounding-box regression loss for coordinating regression to refine the final bounding boxes of the detected objects.

Loss Function for Classification and B-Box Regression

The spatial features extracted after the convolution layer are fed to a network which has two tasks: classification (cls) and regression (reg) by calculating a loss function in each epoch which is back propagated to adjust the final weights of the R-CNN output layer until reaching to a reasonable loss. The loss function measures the compatibility between

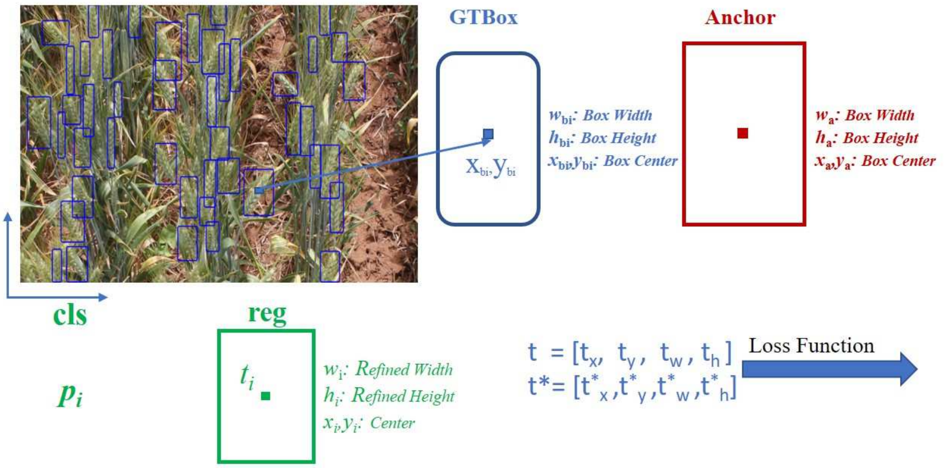

Figure S2.4 Bounding Box Regression to generate loss function.

the prediction and the ground truth label as shown in Figure S2.4. As shown in Equation 3, Intersection of Union (IoU) defines different scores for the overlapping of anchors with ground truths based on the defined thresholds. The loss function is calculated as follows:

$$L(p_i, t_i) = \frac{1}{N_{cls}} \sum_i L_{cls}(p_i, p_i^*) + \gamma \frac{1}{N_{reg}} \sum_i p_i^* L_{reg}(t_i, t_i^*). \tag{3}$$

Here, i is an anchor's index, p_i is the probability of an anchor being an object, and p_i^* is theL ground truth label representing the bounding box coordinates of an anchor. It is set to 1 for the positive anchor and 0 for a negative anchor. Also, t_i^* is the ground-truth box associated with a positive anchor, and L_{cls} is the classification log loss over two classes (object or non-object). The two terms are normalized by N_{cls} and $Nreg$ and weighted by a balancing parameter $\gamma = 3.0$ for RPN and $\gamma = 1.0$ for the detector for smooth L1 loss specified by [7]. Here, $L_{cls}(p_i, p_i^*) = -log(p_i)_{p_i^*}$ is log loss for true class p_i^*. The regression loss in R-CNN is applied by the following equation as defined in [7]:

$$L_{reg}(t_i, t_i^*) = R(t_i - t_i^*), \tag{4}$$

where R is the robust loss function (smooLth L1), and p_i and t_i respectively, are the outputs of the *cls* and *reg* layers. The output of the regressor determines a refined bounding-box as the final output. The output of the classification sub-network is a probability p indicating whether the predicted box contains an object (1 for foreground object) or it is from the background (0 for no object).

For bounding box regression in RPN, we adopt the parameterizations of the four coordinates of ground-truth box $t_i^* = [t_x^*, t_y^*, t_w^*, t_h^*]$ based on the approach of bL[8]. Figure S2.4

shows the GTBox and anchor's properties. Their corresponding mathematical formulation is given by Equation 5 below:

$$
\begin{aligned}
t_x &= (x_i - x_a)/w_a, \\
t_y &= (y_i - y_a)/h_a, \\
t_w &= \log(w_i/w_a), \\
t_h &= \log(h_i/h_a), \\
t_x^* &= (x_{bi} - x_a)/w_a, \\
t_y^* &= (y_{bi} - y_a)/h_a, \\
t_w^* &= \log(w_{bi}/w_a), \\
t_h^* &= \log(h_{bi}/h_a)
\end{aligned}
\tag{5}
$$

We use the publicly available Python implementation of Faster R-CNN, which implements joint approximate training as shown in Figure S2.1. During training, images are horizontally flipped with probability 0.5 to generate more training datasets. No other data augmentation is used. We used an initial learning rate of 10^{-3} which is then reduced by a factor of 10 every 200 epochs. The training was performed for 400 epochs. Initial weight of the CNN is adjusted with 5.0×10^{-4} along with learning using Stochastic Gradient Descent with momentum. The momentum value is set to 0.9. This allows to train Faster R-CNN with any other optimizer without bumping into any big problem like the symmetry when back-propagation. The learning rate multiplier of 0.2 is used for all biases in the network. This, essentially, trains the biases with twice the current learning rate.

Author details

References

1. Krizhevsky, A., Sutskever, I., Hinton, G.E.: Imagenet classification with deep convolutional neural networks. Advances in Neural Information Processing Systems 25, 1097–1105 (2012)
2. Simonyan, K., Zisserman, A.: Very deep convolutional networks for large-scale image recognition. CoRR **abs/1409.1556** (2014)
3. Everingham, M., Eslami, S.M.A., Van Gool, L., Williams, C.K.I., Winn, J., Zisserman, A.: The pascal visual object classes challenge: A retrospective. International Journal of Computer Vision **111**(1), 98–136 (2015)
4. Fei-Fei, L., Fergus, R., Perona, P.: One-shot learning of object categories. IEEE Transactions on Pattern Analysis and Machine Intelligence **28**(4), 594–611 (2006)
5. Canziani, A., Paszke, A., Culurciello, E.: An analysis of deep neural network models for practical applications. CoRR **abs/1605.07678** (2016)
6. Ren, S., He, K., Girshick, R., Sun, J.: Faster R-CNN: Towards real-time object detection with region proposal networks. IEEE Transactions on Pattern Analysis and Machine Intelligence **39**(6), 1137–1149 (2017)
7. Girshick, R.: Fast R-CNN. In: 2015 IEEE International Conference on Computer Vision (ICCV), pp. 1440–1448 (2015)
8. Girshick, R., Donahue, J., Darrell, T., Malik, T.: Rich feature hierarchies for accurate object detection and semantic segmentation. In: 2014 IEEE Conference on Computer Vision and Pattern Recognition, pp. 580–587 (2014)
